# Supplementary material for: Resolving the full spectrum of human genome variation using Linked-Reads
Source: Genome Res. 2019 Apr;29(4):635–45. doi: 10.1101/gr.234443.118 (PMC6442396; doi:10.1101/gr.234443.118)
Supplement: Supplemental Material [file supp_gr.234443.118_Supplemental_longranger-2.2.zip › longranger-2.2.tar.bz2!/lr2.2-public/lib/go/src/code.google.com/vitess/docs/user-guide/upgrading.html]

Vitess / Upgrading


Toggle navigation

- Overview
- Guides
- Reference
- Blog
- GitHub

- #### Overview

  - What is Vitess
  - Scaling MySQL with Vitess
  - Key Concepts
- #### Getting Started

  - Run Vitess on Kubernetes
    - Custom Docker Build
  - Run Vitess Locally
- #### User Guide

  - Introduction- Client Libraries- Backing Up Data- Reparenting
        - Schema Management
        - VSchema Guide
        - Schema Swap (Tutorial)
        - Sharding
          - Horizontal Sharding (Tutorial, automated)
          - Horizontal Sharding (Tutorial, manual)
          - Sharding in Kubernetes (Tutorial, automated)
          - Sharding in Kubernetes (Tutorial, manual)
        - Vitess Sequences
        - MySQL Server Protocol
        - Vitess and Replication
        - Update Stream
        - Row Based Replication
        - Topology Service
        - Transport Security Model
        - Launching
          - Scalability Philosophy
          - Production Planning
          - Server Configuration
          - 2PC Guide
          - Troubleshooting
        - Upgrading
- #### Reference Guides

  - Vitess API- vtctl Commands
- #### Other Resources

  - Presentations- Blog- Roadmap
- #### Contributing

  - Contributing to Vitess- GitHub Workflow- Code Reviews
- GitHub

# Upgrading

- #### Overview

  - What is Vitess
  - Scaling MySQL with Vitess
  - Key Concepts
- #### Getting Started

  - Run Vitess on Kubernetes
    - Custom Docker Build
  - Run Vitess Locally
- #### User Guide

  - Introduction- Client Libraries- Backing Up Data- Reparenting
        - Schema Management
        - VSchema Guide
        - Schema Swap (Tutorial)
        - Sharding
          - Horizontal Sharding (Tutorial, automated)
          - Horizontal Sharding (Tutorial, manual)
          - Sharding in Kubernetes (Tutorial, automated)
          - Sharding in Kubernetes (Tutorial, manual)
        - Vitess Sequences
        - MySQL Server Protocol
        - Vitess and Replication
        - Update Stream
        - Row Based Replication
        - Topology Service
        - Transport Security Model
        - Launching
          - Scalability Philosophy
          - Production Planning
          - Server Configuration
          - 2PC Guide
          - Troubleshooting
        - Upgrading
- #### Reference Guides

  - Vitess API- vtctl Commands
- #### Other Resources

  - Presentations- Blog- Roadmap
- #### Contributing

  - Contributing to Vitess- GitHub Workflow- Code Reviews

# Upgrading a Vitess Installation

This document highlights things to look after when upgrading a Vitess production installation to a newer Vitess release.

Generally speaking, upgrading Vitess is a safe and and easy process because it is explicitly designed for it. This is because in YouTube we follow the practice of releasing new versions often (usually from the tip of the Git master branch).

## Compatibility

As of June 2016 we have not defined formal compatibility guarantees we will promise for future open-source releases. In general, we plan to follow industry standards e.g. compatibility when upgrading to a newer minor version is guaranteed. Upgrades to a higher major may require manual configuration changes and will be mentioned in the changelog.

## Upgrade Order

We recommend to upgrade the different components in the following order:

- vtctld
- vtgate
- vttablet

## Canary Testing

Within the vtgate and vttablet components, we recommend to canary single instances, keyspaces and cells. Upgraded canary instances can "bake" for several hours or days to verify that the upgrade did not introduce a regression. Eventually, you can upgrade the remaining instances.

## Rolling Upgrades

We recommend to automate the upgrade process with a configuration management software. It will reduce the possibility of human errors and simplify the process of managing all instances.

As of June 2016 we do not have templates for any major open-source configuration management software because our internal upgrade process is based on a proprietary software. Therefore, we invite open-source users to contribute such templates.

Any upgrade should be a rolling release i.e. usually one tablet at a time within a shard. This ensures that the remaining tablets continue serving live traffic and there is no interruption.

## Upgrading the Master Tablet

The *master* tablet of each shard should always be updated at last in the following manner:

- verify that all *replica* tablets in the shard have been upgraded
- reparent away from the current *master* to a *replica* tablet
- upgrade old *master* tablet

Contact: vitess@googlegroups.com  **·**  
Announcements  **·**  
© 2017 Vitess powered by Google Inc
